# Desorption of Furfural from Bimetallic Pt-Fe Oxides/Alumina Catalysts

**DOI:** 10.3390/ma7010527

**Published:** 2014-01-20

**Authors:** Gloria Lourdes Dimas-Rivera, Javier Rivera de la Rosa, Carlos J. Lucio-Ortiz, José Antonio De los Reyes Heredia, Virgilio González González, Tomás Hernández

**Affiliations:** 1 Universidad Autónoma de Nuevo León, UANL, Facultad de Ciencias Químicas, Ave. Universidad S/N, Cd. Universitaria, San Nicolás de los Garza, N.L. 64451, Mexico; E-Mails: gloria.dimasrvr@uanl.edu.mx (G.L.D.-R.); tomas.hernandezgr@uanl.edu.mx (T.H.); 2 Universidad Autónoma Metropolitana Iztapalapa, Departamento de Ingeniería Química, Mexico, D.F. 09340, Mexico; E-Mail: jarh@xanum.uam.mx; 3 Universidad Autónoma de Nuevo León, UANL, Centro de Innovación, Investigación y Desarrollo en Ingeniería y Tecnología (CIIDIT), Km 10 de la nueva carretera al Aeropuerto Internacional de Monterrey, PIIT Monterrey, Apodaca, Nuevo León 66600, Mexico; E-Mail: virgilio.gonzalezgnz@uanl.edu.mx

**Keywords:** furfural, bimetallic, Pt-Fe, desorption, impregnation, γ-Al_2_O_3_

## Abstract

In this work, the desorption of furfural, which is a competitive intermediate during the production of biofuel and valuable aromatic compounds, was studied using pure alumina, as well as alumina impregnated with iron and platinum oxides both individually and in combination, using thermogravimetric analysis (TGA). The bimetallic sample exhibited the lowest desorption percentage for furfural. High-resolution transmission electron microscopy (HRTEM) imaging revealed the intimate connection between the iron and platinum oxide species on the alumina support. The mechanism of furfural desorption from the Pt-Fe/Al_2_O_3_ 0.5%-0.5% sample was determined using physisorbed furfural instead of chemisorbed furfural; this mechanism involved the oxidation of the C=O group on furfural by the catalyst. The oxide nanoparticles on γ-Al_2_O_3_ support helped to stabilize the furfural molecule on the surface.

## 1. Introduction

Furfural can be transformed into several important chemical products by hydrogenation, oxidation, reductive amination, decarboxilation, nitration and condensation [1,2]. For example, furfural can be decarbonylated to yield furan, which is used to synthesize valuable aromatic compounds [3,4]. Also, furfurals are important intermediates in the sequences that are being investigated to produce biofuels [1–7]. Therefore, it is important to study furfural adsorption over catalysts to analyze the interaction between the molecule and the surface of the catalyst. Moreover, the chemical steps involved in heterogeneous catalysis imply reactive adsorption, reaction over the surface of the catalyst, and product desorption towards the reacting environment [8].

Bimetallic catalysts have garnered considerable interest and continued investigation because they often exhibit improved catalytic activity and selectivity in heterogeneous reactions relative to their monometallic counterparts [9–11]. In Addition, to understand the origins of the novel catalytic properties, bimetallic surfaces show electronic and chemical properties that are distinct from those of their parent metals, which offer enhanced selectivity, activity, and stability [12]. Furfural is hydrogenated to obtain furfuryl alcohol using the bimetallic Pt/Sn and Pt/Ge catalysts supported on silica [13]. Notably, both metals interact with the surface support but remain unaffected by the specific area, acidity or metal dispersion [14] because the interaction between the metal and the support or promoter may facilitate the adsorption of reactants, contributing to the high furfural conversion and the high selectivity for furfuryl alcohol formation [15]. Adding FeOx to a γ-alumina support enhanced the catalytic activity of pure γ-alumina [16]. Incorporating Fe nanoparticles as a second metal on the alumina improves the sorption/catalytic characteristics with other nanoparticles, such as Pd and Pt [17–20]. In this work, a bimetallic Fe-Pt oxide/γ-alumina catalyst has been synthesized to test its desorption interactions with furfural.

## 2. Results and Discussion

### 2.1. N_2_ Physisorption

The N_2_ adsorption–desorption isotherms for the samples at −196 °C exhibit type IV isotherms, according to the IUPAC classifications, with a typical hysteresis loop due to the capillary condensation of nitrogen into the mesopores [21–23] (data not shown). The specific surface area of the alumina and the catalysts after calcinating at 600 °C are presented in Table 1. The γ-alumina decreases in specific surface area from 244.15 to 195.88 m^2^/g after calcination at 600 °C. In comparison with the uncalcined alumina, the Fe-impregnated catalyst the area decreases just 10%, but when it is impregnated with Pt it decreases 27%; this is due to the Pt particles which block more pores than Fe oxide particles and a stronger interaction of Pt particles with the γ-alumina can be suspected. The bimetallic catalyst has a lower surface area than the rest of the catalysts due to the higher amount of Pt and Fe particles together that have blocked the alumina pores [23–26]. The interactions between the metals and the catalyst can be observed in temperature-programmed reduction (TPR). However, after calcination, the alumina has a lower surface area than the Fe-impregnated catalyst because the impregnated particles prevent the alumina from sintering or compacting, forming more pores in the surface.

### 2.2. SEM and HRTEM Images

**Figure 1.**
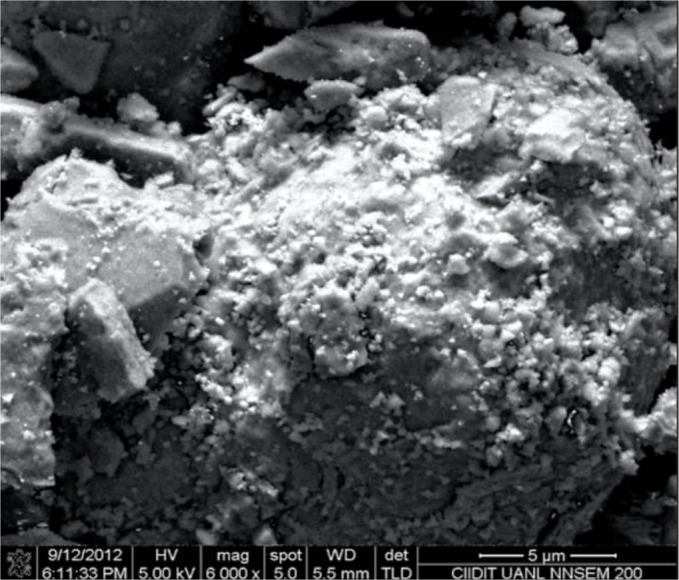
SEM image of Pt-Fe/Al_2_O_3_ 0.5%-0.5%.

**Figure 2.**
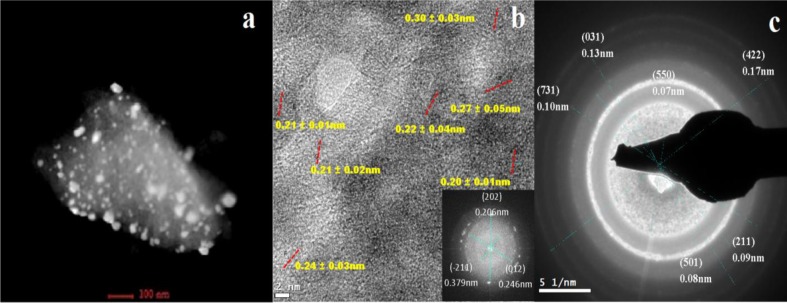
(**a**) STEM image of a grain of Al_2_O_3_ with Pt-Fe oxide nanoparticle; (**b**) high-resolution transmission electron microscopy (HRTEM) image and its Fourier transformed micrograph and (**c**) select area diffraction pattern (SADP) of the same sample.

Figure 1 presents the scanning electron photomicrographs for the samples containing platinum and iron on alumina (Pt-Fe/Al_2_O_3_ 0.5%-0.5%). The Scanning electronic microscopy (SEM) image reveals that the nanoparticles are dispersed on the alumina surface. Several of these nanoparticles are agglomerated, forming 300 to 600 nm structures, while the smaller individual nanoparticles are ~30 nm tetragonal prisms and they are observed on the contours of the alumina grains. Figure 2a displays a scanning transmission electron microscopy (STEM) image of an individual alumina grain with nanoparticles deposited on its surface. More nanoparticles are observed as individuals rather than in aggregates. The flat faces of some of the nanoparticles are very clear, forming almost perfect tetragonal prisms from 10 to 60 nm in size. The other nanoparticles have an ellipsoid shape with slightly deformed and softened contours. These types of nanoparticles range in size from 5 nm individuals to 26 nm agglomerates. The tetragonal nanoparticles are associated with the platinum oxide species [27], while the ellipsoid shapes are associated with the iron oxide species [16,28–30]. Although a wide particle size distribution was achieved, good dispersion was obtained.

Figure 2b provides a high-resolution TEM (HRTEM) image and its Fourier-transformed micrograph (FTM) inset. Perpendicular lines are drawn to identify the interplanar distances (IDs) in the micrographs, and the average is reported. The structures are identified using the Joint Committee on Powder Diffraction Standards (JCPDS). The 0.30 ± 0.03 nm ID is correlated with the (220) plane of the magnetite phase (Fe_3_O_4_), according to JCPDS 82-1533, or the (220) plane of the maghemite phase (γ-Fe_2_O_3_), according to JCPDS 24-0081. The 0.27 ± 0.05 nm ID is correlated with the (221) plane of the magnetite phase (JCPDS 24-0081) or the (104) plane of the hematite phase (α-Fe_2_O_3_), according to JCPDS 86-0550; the 0.22 ± 0.04 nm ID is correlated with the (113) plane of the hematite phase. The two IDs at 0.21 ± 0.03 nm and 0.21 ± 0.02 nm are correlated with the (110) plane of the PtO (JCPDS 01-085-0714), the 0.24 ± 0.03 nm ID corresponds to the (012) plane of the Pt_6_O_8_ species (JCPDS 96-100-8966) and the 0.20 ± 0.01 nm ID is correlated to the (202) plane of the hematite phase. Different platinum and iron oxides are intimately connected on the alumina support. In the FTM, the (202) and (012) planes for hematite and Pt_6_O_8_ are also identified. Figure 2c displays the select area diffraction pattern (SADP) as the rings for the (422) plane for magnetite and the (731) plane for hematite. The (031) plane corresponds to the PtO_2_ phase (JCPDS 01-073-2361), and the (211) plane corresponds to the α-PtO_2_ phase (JCPDS 00-038-1355).

### 2.3. FT-IR Catalyst Studies

**Figure 3.**
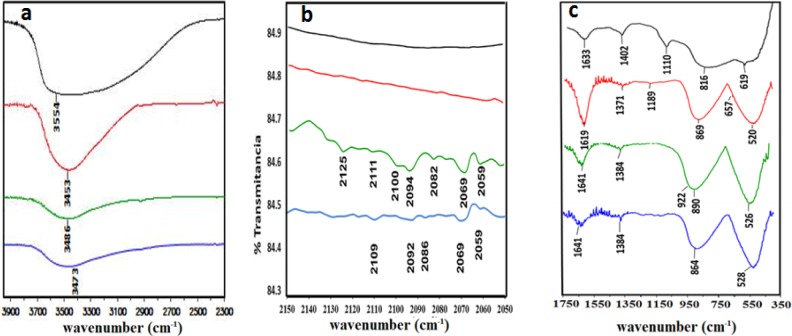
FT-IR spectra in the (**a**) 2300–3900 cm^−1^; (**b**) 2050–2150 cm^−1^ and (**c**) 350–1750 cm^−1^: regions for the alumina (—), Fe/Al_2_O_3_ 0.5% (—), Pt/Al_2_O_3_ 0.5% (—) and Pt-Fe/Al_2_O_3_ 0.5%-0.5% (—) samples.

Figure 3 displays the FT-IR spectra for pure alumina, as well as alumina impregnated with the following: 0.5% Fe, 0.5% Pt and 0.5% of both Pt and Fe. Figure 3a focuses on the 3900 to 2300 cm^−1^ region that contains the bands ascribed to the OH groups from the alumina; this band is very pronounced in the pure alumina, but decreases when metal is present on the support to become much lower when Pt is present. Figure 3b reveals the presence of the CO species adsorbed on the Pt in the range of 2050–2150 cm^−1^ [31,32]. For the pure alumina and Fe/Al_2_O_3_ 0.5% samples do not display any bands in this range. In the Pt/Al_2_O_3_ 0.5% sample, the weak band at 2111 cm^−1^ is attributed to the Pt(III)(CO)_2_ species [32,33]; these species are formed because the Pt adsorbs the CO from the surrounding environment; in the Pt-Fe/Al_2_O_3_ 0.5%-0.5% sample, this signal is shifted toward a lower energy (2109 cm^−1^). There is a series of typical signals attributed to CO species adsorbed on platinum [31–35] in both of the samples containing platinum; some of these signals are observed at the same wavenumber, while others are slightly shifted.

From 350 to 1750 cm^−1^, as displayed in Figure 3c, the pure alumina band at 1633 cm^−1^ is characteristic of the H–O bending mode due to the deformed vibration mode of chemisorbed water [36]. For the samples containing platinum, this band is shifted to 1641 cm^−1^ due to the higher vibrational coupling energy of the H–O functional group. In contrast, this signal was detected at a lower energy (1619 cm^−1^) for the Fe/Al_2_O_3_ sample. In this case, the interaction between the oxide species and the O–H functional groups may be affected by the Brønsted acidic sites on the alumina. The bands arising from the pure alumina sample at 1408 and 1110 cm^−1^ may be attributed to the remnants of the commercial alumina precursors [16,37]. According to the current literature, the presence of Pt (II) is indicated by the signal at ~922 cm^−1^ [33], but this band overlaps with a band assigned to the alumina. The stronger, broader bands at 816 cm^−1^ and 619 cm^−1^ correspond to the vibrations of the AlO_4_ tetrahedra and the AlO_6_ octahedra, respectively, in the pure γ-Al_2_O_3_ sample [16,37]. The impregnation with the oxides shifts the tetrahedral band to a higher vibrational energy, while the octahedral band migrates to lower values; therefore, there is a stronger interaction with the tetrahedral sites. The presence of the Pt oxide species generates shoulder peaks at low energies (426 and 410 cm^−1^), and the iron oxide species has only a weak shoulder at 657 cm^−1^ that may be correlated to the maghemite phase [38].

### 2.4. Temperature Programmed Reduction

**Figure 4.**
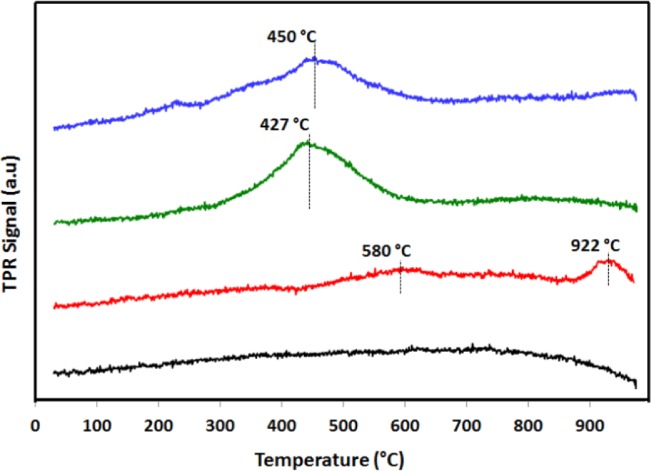
Temperature-programmed reduction (TPR) of alumina (**—**); Fe/Al_2_O_3_ 0.5% (**—**); Pt/Al_2_O_3_ 0.5% (**—**) and Pt-Fe/Al_2_O_3_ 0.5%-0.5% (**—**) substrates.

Figure 4 presents the TPR traces for the bi- and monometallic γ-Al_2_O_3_ catalysts, calcined at 600 °C. The calcined γ-Al_2_O_3_ does not have a reduction peak. However, two reduction peaks at 580 and 922 °C can be observed for Fe/Al_2_O_3_. The first signal is attributed to the reduction of the Fe_2_O_3_ to Fe_2_O_3_ and the second peak is attributed to the reduction of the Fe_2_O_3_ to FeO + Fe [39–41]. The TPR profile of the monometallic Pt catalysts reveals a reduction peak at 427 °C corresponding to Pt oxy- or hydroxychlorinated species being reduced to elemental Pt [42,43]. A reduction peak similar to that for the Pt catalyst but less intense is observed for the bimetallic Pt-Fe catalysts. Researchers have observed that the presence of Pt in bimetallic catalysts promotes the reduction of the other metal; therefore, the reduction peak is shifted toward lower temperatures due to increased metal-metal versus metal-support interactions [42,43]. For example, the Ge oxide species are reduced at 600 °C, but Pt incorporation lowers the reduction temperature to 315 °C [42]. Another interesting feature of the bimetallic oxide TPR results is the presence of only one reduction signal, compared to the two peaks observed for the Fe catalyst. The appearance of a single TPR peak shows that the reduction of two metal oxides is essentially simultaneous, and an alloy is formed. This phenomenon is a common evidence to judge the formation of alloy in the bimetallic catalyst [44,45]. The TPR results agree with HRTEM data because both indicate that there is a strong interaction between the Pt and Fe, which is characteristic of alloys. TPR results also agree with HRTEM; different Pt and Fe species are present with different shapes, sizes and structures across the surface of the catalyst. The Fe is present as maghemite, magnetite and hematite, while Pt is observed as oxides and coordinated species. In addition, the peak for the Pt-Fe/A_2_O_3_ catalyst is slightly less pronounced than the peak for the Pt/Al_2_O_3_. The reduction temperature for this alloy is lower than Fe reduction temperature, indicating that the alloy particle size decreases due to the increasing particle dispersion that reduces the H_2_ consumption, according to reports by Pojanavaraphan *et al.* [46].

### 2.5. TGA Desorption

**Figure 5.**
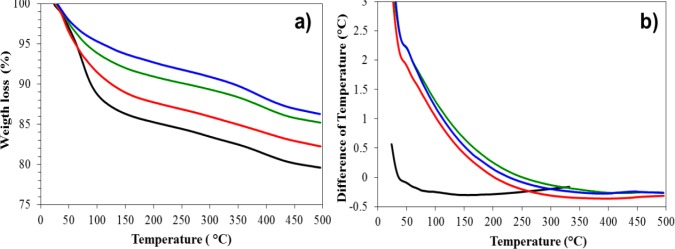
(**a**) Thermogravimetric analysis (TGA) and (**b**) DTA of furfural on different substrates: alumina (**—**), Fe/Al_2_O_3_ 0.5% (—), Pt/Al_2_O_3_ 0.5% (—) and Pt-Fe/Al_2_O_3_ 0.5%-0.5% (**—**).

Figure 5a displays the thermogravimetric data in which the pure alumina has a higher furfural desorption than the doped alumina. All the catalysts (with and without furfural impregnation) were analyzed by TGA. For the catalysts without furfural, no water is released; therefore, water does not interfere with the analysis of furfural desorption. In the differential thermal analysis (Figure 5b), a slight endothermic change is observed below 100 °C. However, this signal does not represent a considerable chemical event; instead, it reveals a physical interaction between the catalyst and the furfural. Table 2 lists the steps of the thermogravimetric data with the weight loss percentage (WLP). In the first step, the pure alumina and Fe/Al_2_O_3_ 0.5% samples present a ≥10% WLP. The Pt/Al_2_O_3_ 0.5% sample displays a 6.7% WLP, while the Pt-Fe/Al_2_O_3_ 0.5%-0.5% sample loses only 5%. During the second step, all of the samples lose more than 11%, except for the Pt-Fe/Al_2_O_3_ 0.5%-0.5% sample that loses only 10.8%. Some investigators have determined the furfural desorption in different materials: Zhang *et al.* [4] studied furfural desorption in K doped Pd/Al_2_O_3_ catalysts using programmed temperature up to 600 °C to observe only a single, intense desorption signal at 67 °C; Vorotnikov *et al.* [47] reported that molecular furfural desorption begins around −13 °C to complete by 187 °C after peaking at ~92 °C using temperature-programmed desorption (TPD) data, and the breadth of the desorption peak indicated that different adsorption states also desorb furfural, generating different molecular decomposition reactions after increased exposure; Pang *et al.* [48] found that furfural molecular desorption occurs at ~102 °C. During our TGA tests, the iron and platinum oxides in the bimetallic catalyst presented a higher furfural adsorption strength because they release approximately 13.7% of the furfural at 495 °C, while other samples released more than 14.7%. The DTA results do not indicate that there was any chemical event during the desorption process; indicating that the furfural adsorption states are stable intermediates that prevent the decomposition.

Equation (1) is a kinetic model of an organic molecule desorption:

dα/dt=k(T)f(α) (1)

where α = (*w*_0_ – *w_t_*)/(*w*_0_ – *w_f_*), *w_t_* is the mass at time *t; w*_0_ is the initial mass and *w_f_* is the mass at the last temperature from the test, 500 °C. The kinetic constant follows the Arrhenius equation:

$$\text{d}\alpha /\text{d}tk(T)=k_{0}\text{ exp}(-E_{a}/\text{R}T)$$ (2)

where *k*_0_ is the pre-exponential factor; R is the gas constant; *T* is the temperature and *E_a_* is the apparent activation energy. The form of *f*(α) can be expressed as follows:

$$f(\alpha )={\left(1-\alpha \right)}^{n}\alpha^{m}{\left[\text{ln}(1-\alpha )\right]}^{p}$$ (3)

where the exponential values of *m* and *p* are considered equal to zero, according to other desorption–degradation studies of organic samples [49]. Assuming that the reactions engaged during the desorption process are first order, Equation (1) can be written as follows:

$$\text{d}\alpha /\text{d}t=k_{0}\text{ exp}(-E_{a}/\text{R}T)(1-\alpha )$$ (4)

Using the heating rate expression is as follows:

N=dT/dt (5)

The equation can be resolved into the differential Equation (4):

$$\text{ln}[-\text{ln}(1-\alpha )]={\text{lnk}}_{0}-(E_{a}/\text{R}T)$$ (6)

This method is known as Broido’s method [50].

**Figure 6.**
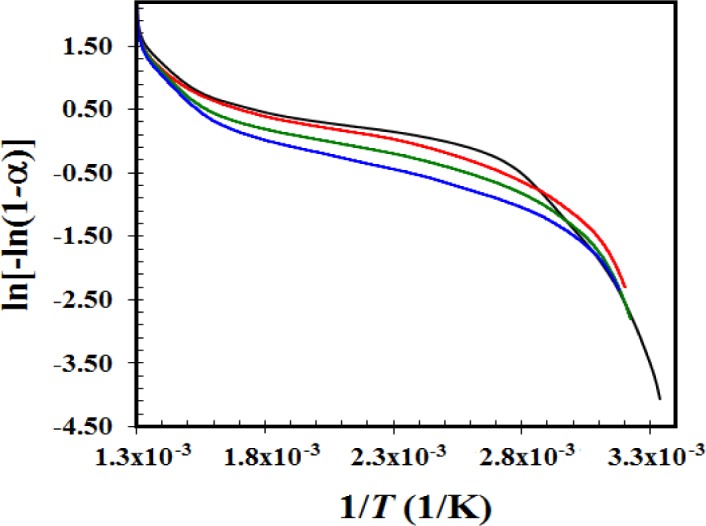
Experimental data for alumina (**—**), Fe/Al_2_O_3_ 0.5% (**—**), Pt/Al_2_O_3_ 0.5% (**—**) and Pt-Fe/Al_2_O_3_ 0.5%-0.5% (**—**) samples treated according Equation (6).

Figure 6 displays the experimental TGA data treated using Equation (6), and the activation energy values for each step are reported in Table 2. The low activation energies (less than 10 kcal/mol ≈ 41.84 kJ/mol) indicate that a transport mechanism is more prevalent than the entirely reactive mechanism [50]. The first step for pure alumina has activation energy of 39.10 kJ/mol. This value decreases when platinum or iron are incorporated into the alumina, with values more than 50% lower when both platinum and iron are incorporated. For the second step, the activation energies for all of the samples are below 8 kJ/mol, indicating the mechanism is entirely physical. During the third step, the activation energy of every sample exceeds 30 kJ/mol except for the bimetallic oxide sample (8.20 kJ/mol). Activation energies near 40 kJ/mol suggest a mechanism that involves breaking chemical bonds but are still far below the values needed for pyrolysis degradation [50]. The sample containing platinum and iron oxides display a physical-type desorption throughout the entire temperature range. Sitthisa *et al.* [51] present a model of the Langmuir-Hinshelwood kinetics for the hydrogenation of furfural over Cu/SiO_2_; the equilibrium adsorption constant (that considers adsorption and desorption) falls from 0.29 to 0.08 torr^−1^ (−72%) from 230 to 290 °C. Even the test is different for our Pt-Fe/Al_2_O_3_ 0.5%-0.5% sample; the desorption constant never falls more than 14% from 25 to 495 °C (see Table 2). From the second step to the third, the bimetallic catalyst displays a slight increase (3%) in the *k*_0_ constant, indicating there are different interactions between the molecule and the support during each stage [47]. During the second step, the slower furfural release and the smaller *k*_0_ reveal a more stable interaction.

### 2.6. FT-IR Spectra of Furfural Desorption on Pt-Fe/Al_2_O_3_ 0.5%-0.5%

**Figure 7.**
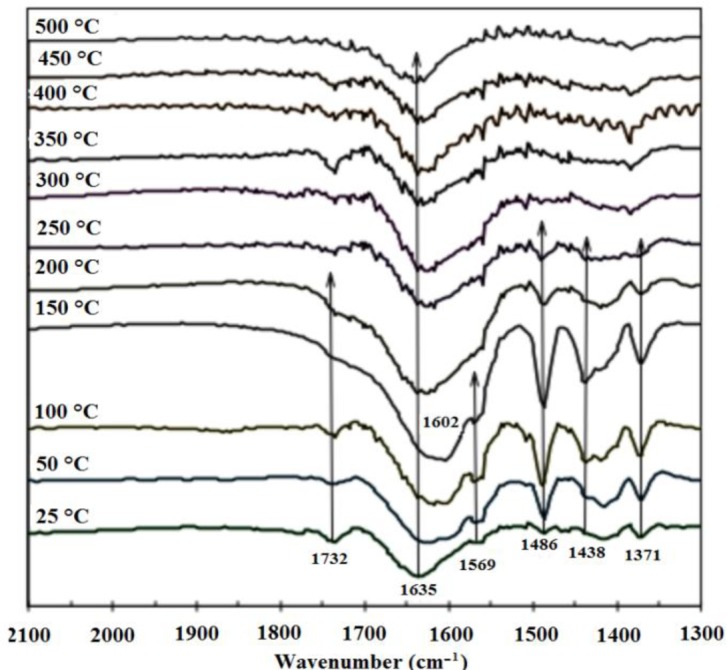
FT-IR spectra furfural desorption at different temperatures in the Pt-Fe/Al_2_O_3_ 0.5-0.5% sample.

Because the bimetallic sample contains the most stable furfural species during desorption, the FT-IR spectra are studied at different temperatures during furfural desorption. Furfural contains two O atoms, one in the carbonyl group and one in the aromatic ring. These two atoms that are the possible adsorption points in furfural, and they are 2.809 Å apart. At the molecular level, the adsorption configurations of furfural on the surfaces can be divided into three types: (a) parallel-ring adsorption, where the furfural molecule is roughly parallel to the surface; (b) carbonyl adsorption, where the C=O bond is involved in the interaction with the surface and (c) perpendicular adsorption, where the furfural molecule is approximately perpendicular to a crystalline surface plane [51]. The strong interaction of the O atom in the carbonyl group on furfural and the other aldehydes on different surfaces has been studied, and their signals in the FT-IR spectra indicate weak energy interactions, generally from 1300 to 2100 cm^−1^. These aldehydes tend to form surface intermediates in which only the carbonyl O can be adsorbed on one metal atom (*i.e.*, η^1^(O)) or both C and O interact with the surface (*i.e.*, η^2^(C,O)). For example, while the η^2^(C,O) state is preferred on clean Pd surfaces, the η^1^(O) state is preferred when oxygen is present [52,53]. Therefore, the adsorption of η^1^(O) may be more likely in our samples. Figure 7 displays the FT-IR spectra for furfural desorbing at different temperatures from the Pt-Fe/Al_2_O_3_ 0.5%-0.5% sample. The band at 1635 cm^−1^ in the pure catalyst sample is associated with the OH groups of the ɣ-alumina support, but this band is widened, forming a peak at 1602 cm^−1^ with a shoulder at 1569 cm^−1^ from 25 to 200 °C in the furfural-adsorbed samples. These bands are attributed to chemisorbed furfural, while the band at 1732 cm^−1^ is associated with the physisorbed furfural [4] that nearly disappears at 300 °C, appears again at 350 °C and tends to disappear again at 450 °C. The bands at 1486 and 1438 cm^−1^ that disappear at 300 °C are associated with the vibrations of the furan ring double bond. The band at 1371 cm^−1^ from 25 to 250 °C may be related to the oxidized η^2^(C,O) (ν_s_(OCO)) species [6], revealing an interaction with oxygen from an oxide species on the catalyst, together with a strong interaction existing between metallic species, according to the TPR and TEM results. This interaction warrants a discussion regarding the type of acidity present in the catalyst because the carbonyl oxygen from furfural is Lewis basic and is therefore capable of sharing a pair of electrons; consequently, Lewis acidic sites must also exist in the catalyst for this bond to be formed.

The liquid impregnated furfural almost completely disappears at 200 °C, but some bands remain detectable from 300 to 450 °C, validating the TGA/DTA data. No band appears at ~1957 cm^−1^ to indicate CO adsorption from thermally decarboxylated furfural [4], revealing the favorably improved stability of furfural on our bimetallic sample.

## 3. Experimental Section

### 3.1. Synthesis of Catalysts

The CATALOX^®^ (Houston, TX, USA) γ-alumina, FeCl_3_ and PtCl_2_ (by Sigma-Aldrich, St. Louis, MO, USA) were mixed in water, the cationic concentration of the solution was adjusted to 0.5 wt% on the alumina and the solution was stirred for 24 h. The water was subsequently evaporated at 80 °C, and the catalyst was calcined at 600 °C. The catalysts were prepared in the following compositions: Fe/Al_2_O_3_ 0.5%, Pt/Al_2_O_3_ 0.5% and Pt-Fe/Al_2_O_3_ 0.5%-0.5%.

### 3.2. Characterization Techniques

The specific surface areas of the samples were determined using N_2_ physisorption at 77.3 K with a Quantachrome-NovaWin2 (Boynton Beach, FL, USA). SEM photomicrographs were obtained with a FEI Nova Nano SEM 200 (FEI, Hillsboro, OR, USA) at 20 kV at a 1 nm resolution. The working distance and energy dispersive spectra (EDS) were collected using an EDAX Genesis XM4 detector for elemental analysis (EDAX Inc, Mahwah, NJ, USA) A FEI Titan G2 30-800 microscope was used for high-resolution transmission electron microscopy (HRTEM) imaging. The FT-IR spectra were measured on a Nicolet 6700 spectrometer (FEI), for samples in mixed KBr disks. The temperature-programmed reduction (TPR) of the catalysts was carried in flowing a 10% H_2_/Ar mixture at 30 mL/min with heating at 5 °C/min. Before the analysis, the catalysts were pretreated in argon at 300 °C for 30 min. The hydrogen consumption was monitored using the thermal conductivity detector with mass spectrometry capabilities using an Altamira Instruments model AMI 90 (Altamira Instruments Inc, Pittsburgh, PA, USA).

For the desorption tests, all catalysts were impregnated with 30% (total weight) of furfural, with the following quantities: alumina; 0.401 g of powder and 0.170 g of furfural (29.77%), Fe/Al_2_O_3_; 0.504 g of powder and 0.218 g of furfural (30.19%), Pt/Al_2_O_3_; 0.401 g of powder and 0.175 g of furfural (30.38%), Pt-Fe/Al_2_O_3_; 0.412 g of powder and 0.178 g of furfural (30.17). The excess of furfural was desorbed at room temperature for 10 min. The catalysts were then analyzed by TGA/DTA using a TA Instruments SDT-2960 thermal analyzer, with a temperature slope of 5 °C/min, from 25 to 500 °C in N_2_ with a flow of 100 mL/min.

## 4. Conclusions

The desorption of furfural on samples of pure alumina and samples with 0.5% Fe and 0.5%–5% Pt oxides was studied; the bimetallic sample exhibited the best thermal properties and resistance toward furfural desorption. The Pt oxide species, such as PtO and Pt_6_O_8_, were identified in the HRTEM images, and magemite, maghemite and hematite phases of iron oxide were also identified. Both types of crystalline oxides were intimately connected on the alumina support. The FT-IR studies indicated that impregnating both metallic oxides affected the tetrahedral (AlO_4_) and octahedral (AlO_6_) sites of the alumina. The TPR results revealed that Fe and Pt are present over the catalyst surface as iron oxides and Pt oxy- or hydroxychlorinated species. The TPR results were in agreement with the HRTEM analysis because both suggested that a strong interaction characteristic of alloys was present between Pt and Fe and that different Pt and Fe species are present with different shapes, sizes and structures across the catalyst surface. For the pure alumina and the samples impregnated both single and bimetallic Fe and Pt oxides, the mechanism of desorption from approximately 113 °C to approximately 370 °C tended to be more like physisorption. The mechanism for the temperatures below 113 °C and above 370 °C resembled chemical desorption, based on the activation energy values obtained during the TGA tests. The Pt-Fe/Al_2_O_3_ 0.5%-0.5% sample exhibited no significant changes in the low energies of activation over the complete temperature range, demonstrating a more homogenous mechanism of desorption. The FT-IR spectra at different temperatures during the furfural desorption from the Pt-Fe/Al_2_O_3_ 0.5%-0.5% sample revealed both chemisorption and physisorbtion interactions, but the aldehyde radical of furfural and the specific η^2^(C,O) interaction with the surface revealed the formation of oxide species (OCO) with the sample. The bimetallic Fe-Pt oxide sample is a good catalyst that stabilizes the adsorbed furfural molecule for subsequent reactions.

## Figures and Tables

**Table 1.** Textural properties and weight percent of metals in the catalysts.

Sample	(%)		Surface area (m^2^/g)	Average pore diameter (nm)	Average pore volume (cc/g)
	Fe	Pt			
Alumina*	–	–	196	8.83	0.43
Fe/Al_2_O_3_ 0.5%	0.44	–	218	8.40	0.46
Pt/Al_2_O_3_ 0.5%	–	0.60	177	9.31	0.41
Pt-Fe/Al_2_O_3_ 0.5%-0.5%	0.43	0.47	169	9.56	0.40

* Calcination at 600 °C

**Table 2.** Thermogravimetric data at collected at 10 °C/min under N_2_ (15 mL/min) and the activation energy according to Equation (6).

Sample	Step	Range of Temperature (°C)	WLP (%) *	*E_a_* (kJ/mol)	*k*_0_ (s^−1^)
Alumina	1st	26–113	13.0	39.10	2.74 × 10^5^
	2nd	113–376	18.0	6.11	6.14
	3rd	376–495	20.3	34.30	1.11 × 10^3^

Fe/Al_2_O_3_ 0.5%	1st	26–121	10.0	21.90	7.69 × 10^2^
	2nd	121–372	15.5	7.06	7.07
	3rd	372–496	17.7	31.87	5.77

Pt/Al_2_O_3_ 0.5%	1st	27–113	6.7	24.72	2.92 × 10^5^
	2nd	113–365	12.0	7.28	6.02
	3rd	365–496	14.8	36.65	1.58 × 10^3^

Pt-Fe/Al_2_O_3_ 0.5%-0.5%	1st	29–114	5.0	17.00	12.0
	2nd	114–370	10.8	5.63	0.357
	3rd	370–495	13.7	8.20	0.543

* = Accumulated
